# Probiotic *Lactobacillus* sp. Strains Inhibit Growth, Adhesion, Biofilm Formation, and Gene Expression of Bacterial Vaginosis-Inducing *Gardnerella vaginalis*

**DOI:** 10.3390/microorganisms9040728

**Published:** 2021-03-31

**Authors:** Zhixiang Qian, Hui Zhu, Dan Zhao, Ping Yang, Fei Gao, Chunyi Lu, Yu Yin, Shidong Kan, Daijie Chen

**Affiliations:** 1 College of Pharmacy, Shanghai Jiaotong University, Shanghai 200240, China; 820876094@sjtu.edu.cn (Z.Q.); julie19930@sjtu.edu.cn (H.Z.); 20135310341@sjtu.edu.cn (D.Z.); yp_930201@sjtu.edu.cn (P.Y.); feigao@sjtu.edu.cn (F.G.); luchunyi@sjtu.edu.cn (C.L.); yinyuly@sjtu.edu.cn (Y.Y.); chen_lab@sjtu.edu.cn (S.K.); 2 State Key Laboratory of Microbial Metabolism, Shanghai Jiao Tong University, Shanghai 200240, China; 3 Shanghai Institute of Pharmaceutical Industry, China State Institute of Pharmaceutical Industry, Shanghai 201203, China

**Keywords:** *Lactobacillus*, *Gardnerella vaginalis*, biofilm, gene expression, anti-inflammatory

## Abstract

*Gardnerella vaginalis* contributes significantly to bacterial vaginosis, which causes an ecological imbalance in vaginal microbiota and presents with the depletion of *Lactobacillus* sp. *Lactobacillus* supplementation was reported to be an approach to treat bacterial vaginosis. We investigated the applicability of three *Lactobacillus* sp. strains (*Lactobacillus delbrueckii* DM8909, *Lactiplantibacillus plantarum* ATCC14917, and *Lactiplantibacillus plantarum* ZX27) based on their probiotic abilities in vitro. The three candidate *Lactobacillus* sp. strains for bacterial vaginosis therapy showed distinct properties in auto-aggregation ability, hydrophobicity, adhesion to cervical epithelial cells, and survivability in 0.01% hydrogen peroxide. *Lpb. plantarum* ZX27 showed a higher yield in producing short-chain fatty acids and lactic acid among the three candidate strains, and all three *Lactobacillus* sp. strains inhibited the growth and adhesion of *G. vaginalis*. Furthermore, we discovered that the culture supernatant of *Lactobacillus* sp. exhibited anti-biofilm activity against *G. vaginalis*. In particular, the *Lpb. plantarum* ZX27 supernatant treatment decreased the expression of genes related to virulence factors, adhesion, biofilm formation, metabolism, and antimicrobial resistance in biofilm-forming cells and suspended cells. Moreover, *Lactobacillus* sp. decreased the upregulated expression of interleukin−8 in HeLa cells induced by *G. vaginalis* or hydrogen peroxide. These results demonstrate the efficacy of *Lactobacillus* sp. application for treating bacterial vaginosis by limiting the growth, adhesion, biofilm formation, and virulence properties of *G. vaginalis*.

## 1. Introduction

Bacterial vaginosis (BV) is a common infection of the female reproductive tract [1]. Based on Amsel criteria, BV prevalence was up to 8.7% in Beijing (2010), 5.9% in Shandong (2004), and 15.4% in Sichuan (2004) [2]. BV is characterized by the replacement of beneficial lactobacilli and the augmentation of anaerobic pathogenic bacteria, such as *Gardnerella vaginalis* [3,4,5]. *G. vaginalis* is postulated as the initial pathogenic bacteria that adhere to the vaginal epithelium, where it requires the assistance of other anaerobes to promote BV development [6]. It is now accepted that biofilms in BV are strongly associated with *G. vaginalis* [7], and *G. vaginalis*-forming biofilms on the vaginal epithelium contribute to the poor cure rates of antimicrobial therapy [8].

The virulence factors of *G. vaginalis* include vaginolysin and sialidase (encoded by *vly* and *sld*, respectively) that contribute to BV formation [9]. Vaginolysin is a member of the cholesterol-dependent cytolysin family and acts as a hemolysin [10] by creating pores that alter cell integrity and shape, which is thought to disrupt local innate immunity, and thus, promote BV persistence [11]. Sialidase (also known as neuraminidase) is an enzyme that cleaves sialic acid from the terminal glycans of glycoproteins and is also present in cervicovaginal fluid [12]. Sialidase activity is a proposed biomarker for BV [13] and has been independently associated with adverse pregnancy outcomes, including intrauterine infections and preterm births [14].

Clindamycin and metronidazole are the standard drugs for BV [15]. However, their use is associated with antibiotic resistance and a high recurrence of the infection [15,16]. The biofilms of anaerobic pathogenic bacteria in vaginal microorganisms might be responsible for the treatment failure and recurrence of BV [17]. Adjuvant therapy with probiotics was recommended by experts, and some studies investigated the role of probiotics in BV treatment, including the use of probiotics alone and in combination with antibiotics [16]. A recent phase 2b trial reported that *Lactobacillus crispatus* CTV-05 (Lactin-V) significantly reduced BV recurrence [18].

*Lactobacillus* sp. is the dominant species in about 70% of vaginas (26.2% with *L. crispatus*, 6.3% with *Lactobacillus gasseri*, 5.3% with *Lactobacillus jensenii*, and 34.1% with *Lactobacillus iners* in White/Asian women), and could promote vaginal health by suppressing the risk of BV [19]. The prevalence of these community types varies with race and ethnicity, with Black and Hispanic women more frequently hosting *L. iners*-dominant and diverse communities than White women, who more frequently host *L. crispatus*-dominant communities [20]. *L. crispatus* can repress the expression of *vly* and *sld* genes in *G. vaginalis* [9,21], and *Lactobacillus reuteri* RC-14 produced the largest displacement of *G. vaginalis* in the biofilm [22]. *Lactiplantibacillus plantarum* 59 and *Lactobacillus fermentum* 137 were confirmed to exhibit anti-inflammatory effects against *G. vaginalis* and *Candida albicans* and to be capable of influencing the nuclear factor-kappaB signaling pathway [23]. *Lpb. plantarum* ZX27 is a typical probiotic microorganism found in old yogurt, and we previously demonstrated its antimicrobial effects against *G. vaginalis* and *Escherichia coli* [24]. However, to date, no study has investigated the anti-biofilm effect of *Lpb. plantarum* against *G. vaginalis*, and the associated mechanisms remain unknown.

Accordingly, in the present study, we investigated the functions of two *Lpb. plantarum* strains (*Lpb. plantarum* ZX27 and *Lpb. plantarum* ATCC 14917) and one commercial lactic acid bacteria (LAB) strain (*L. delbrueckii* DM8909) and compared the probiotics based on their anti-adhesion, co-culture, and anti-biofilm activities against *G. vaginalis*. We further elucidated the possible mechanisms by evaluating the gene expression of associated parameters, including virulence factors, epithelial adhesion, biofilm formation, metabolism, and antimicrobial resistance of *G. vaginalis*. Finally, we assessed the anti-inflammatory activity of probiotics against *G. vaginalis* and oxidative stress (OS).

## 2. Materials and Methods

### 2.1. Bacterial Strains, Media, Cell Lines, and Growth Conditions

Three *Lactobacillus* sp. (*Lpb. plantarum* ZX27, isolated from Mongolian yogurt; *Lpb. plantarum* ATCC14917, purchased from the Guangdong culture collection center, Canton, Guangdong province, China; and *L. delbrueckii* DM8909, isolated from Dingjunsheng, Inner Mongolia Shuangqi Pharmaceutical Co. Ltd.) were selected to explore the effects of lactobacilli on *G. vaginalis*. ATCC49145 (purchased from the Guangdong culture collection center, Canton, Guangdong province, China) is a vaginolysin-producing strain [10]. *Lactobacillus* sp. and *G. vaginalis* were cultured in deMan, Rogosa, and Sharpe (MRS) broth and brain-heart infusion (BHI) broth supplemented with yeast extract (1%), maltose (0.1%), glucose (0.1%), and horse serum (10%) (BHIs), respectively, at 37 °C under anaerobic conditions in an anaerobic system (Ruskinn Technology, Ltd., Bridgend, UK). HeLa cells, a cervical epithelial cell line, were cultured in Dulbecco’s modified Eagle medium (DMEM; HyClone, Logan, UT, USA), and supplemented with 10% fetal bovine serum (Gibco, Gaithersburg, MD, USA) and 1% antibiotics (final concentrations: 100 units/mL penicillin and 100 μg/mL streptomycin) (Gibco) at 37 °C in a cell incubator (Thermo Fisher Scientific, Waltham, MA, USA) under a 5% CO_2_ atmosphere.

### 2.2. Investigating the Auto-Aggregation Ability, Hydrophobicity, Adhesion Ability to HeLa Cells and Survivability in 0.01% H_2_O_2_ of Three Candidate Strains

All three LAB strains were assessed in terms of the following characteristics: auto-aggregation ability, hydrophobicity, adhesion to HeLa cells, and survivability in a 0.01% H_2_O_2_ environment.

Auto-aggregation assay was adapted from Pessoa et al. [25]. For the auto-aggregation assay, all *Lactobacillus* sp. strains were grown in MRS broth for 12 h. After centrifugation (10,000× *g*, 10 min, at 4 °C), the cell pellets were resuspended, washed twice with 0.01 M phosphate-buffered saline (PBS), and resuspended to 1 × 10^8^ Colony-Forming Unit/mL (CFU/mL) in the same solution. The suspensions were vortexed and incubated at 37 °C for 4 h, and an aliquot (1 mL) from the top of the suspension was carefully removed for assessment of the optical density at 600 nm (OD_600_) using a spectrophotometer (UV-1100; Mapada Instruments Co., Ltd., Shanghai, China). The formula for calculating auto-aggregation is as follows: auto-aggregation (%) = (1 − A_t_/A_0_) × 100%, where A_0_ indicates the absorbance at 0 h, and A_t_ indicates the absorbance at 4 h. The auto-aggregation assay for the three lactobacilli was performed in triplicate.

We used microbial adhesion to hydrocarbons to determine the degree of hydrophobicity, which was adapted from Rong et al. [26]. Xylene was used as the solvent, and lactobacilli strains were prepared similarly to the auto-aggregation assay. The lactobacilli strains were resuspended in a 0.1 M potassium nitrate (pH 6.2) solution and washed twice with 0.01 M PBS. The solution was then adjusted to an OD_600_ of 0.5 A_0_. We mixed 3 mL of each *Lactobacillus* sp. suspension with 1 mL of xylene prior to vortex for 10 s, and incubated them at room temperature (25 °C) for 10 min. The mixtures were then vortexed for 2 min prior to incubation at room temperature (25 °C) for 20 min for phase separation. The aqueous phase was gently removed to measure its absorbance at 600 nm (A_1_). The surface hydrophobicity (%) was calculated as (1 − A_1_/A_0_) × 100%. The hydrophobicity assay for the three lactobacilli was performed in triplicate.

Adhesion to HeLa cells was assayed according to the methods of [27]. Briefly, HeLa cells covering the bottom of a 12-well cell plate were washed twice with pre-warmed PBS. Bacterial cells (10^9^ CFU) were then resuspended in 0.5 mL DMEM and incubated with HeLa cells for 3 h at 37 °C, after which the epithelial cell cultures were removed and washed twice with pre-warmed PBS. Subsequently, 100 μL of trypsin-Ethylene Diamine Tetraacetic Acid (EDTA) (1×; Invitrogen, Carlsbad, CA, USA) was added to each well and incubated for 10 min at 37 °C to lyse the cells. PBS (900 μL) was then added to the solution and mixed, and serial dilutions were performed, followed by incubation at 37 °C for 24 h. The adhesion ratio was calculated by comparing the number of adherent cells to the original bacterial suspension (10^9^ CFU/mL). The number of adhered lactobacilli strains on the HeLa cells was calculated by comparing the number of adherent bacterial cells to the number of HeLa cells (10^6^/well). The adhesion of each of the three lactobacilli was tested in triplicate in three independent experiments.

Stress survival tests were performed in accordance to [27], with a slight modification. Briefly, based on estimations via the OD_600_ measurements, 10^8^ CFU/mL bacterial cells were resuspended in 1 mL of 0.01% H_2_O_2_ and incubated at 37 °C for 90 min. The survival percentage was calculated by comparing the exact CFU (plate counts) before and after their addition to 0.01% H_2_O_2_. PBS was used as a baseline, and the negative control was lactobacilli incubated in PBS. Each strain was tested three times, and each experiment was performed in triplicate. 

### 2.3. Short-Chain Fatty Acids (SCFAs) and Lactic Acid Analysis in Cell-Free Supernatants (CFS)

CFS from the three *Lactobacillus* sp. strains were prepared as described in our previous study [24]. Briefly, *Lactobacillus* sp. was incubated in MRS broth for 24 h in an anaerobic culture device (Ruskinn Technology, Ltd.), followed by centrifugation (8000× *g*, 20 min) and filtering (0.22 μm). An evaluation of SCFAs and lactic acid in the CFS was performed according to the methods of [28]. The CFS (400 μL) was added to 100 μL of 50% aqueous sulfuric acid and 500 μL of diethyl ether. After vortexing and centrifugation (12,000× *g*, 10 min), the extracted SCFAs and lactic acid extracted in the upper layer (diethyl ether) were analyzed by gas chromatography (GC-2010; Shimadzu, Kyoto, Japan), with 2-methylpentanoic acid as the internal standard. The chromatographic conditions were as follows: injection chamber temperature of 220 °C; injection volume of 1 μL; shunting ratio of 5:1; flow rate of 2 mL /min; column temperature of 100 °C reserved for 1 min, 5 °C/min increased to 170 °C, 30 °C/min increased to 230 °C and reserved to 100 °C in 2 min; and flame ionization detector temperature of 250 °C. Three samples of the CFS from the three *Lactobacillus* spp. were tested.

### 2.4. Co-Aggregation Assay

A co-aggregation assay was adapted from Pessoa et al. [25]. For the co-aggregation assay, the *Lactobacillus* sp. suspension was prepared similarly to the auto-aggregation assay. The *G. vaginalis* suspension was prepared and standardized to 1 × 10^8^ CFU/mL in 0.01 M PBS. Each *Lactobacillus* sp. suspension (1 mL) was mixed with the same volume of *G. vaginalis* suspension, and the mixture was vortexed for 10 s prior to gravity sedimentation. Control tubes contained 2 mL of each bacterial suspension alone. The absorbance of the suspensions was read at 600 nm in a spectrophotometer after a 3-h incubation at 37 °C. Co-aggregation was calculated using the following formula: co-aggregation (%) = [(*Ax*+*Ay*)/2−*A* (*x*+*y*)] / [(*Ax*+ *Ay*)/2] %, where *Ax* and *Ay* indicate the absorbance of strains in the control tubes, and *A* (*x* + *y*) indicates the absorbance of the mixtures.

### 2.5. Co-Culture Assay

We used the bacterial co-culture technique described by Jang et al. [29] to evaluate the influence of *Lactobacillus* sp. on *G. vaginalis* growth. *G. vaginalis* (10^4^ CFU/mL) was inoculated into 5 mL of BHI broth in the presence of three *Lactobacillus* sp. strains (at 10^5^, 10^6^, or 10^7^ CFU/mL) for 24 h. The broth absence of *Lactobacillus* sp. was chosen as the control.

We used a DNA rapid extraction kit (Sangon Biotech, Shanghai, China) to extract the total DNA from 1 mL of co-cultured suspension culture, followed by amplification of 1 μL of DNA by real-time qPCR using the SYBR Green Real-Time PCR Master Mix (Toyobo, Osaka, Japan) on a StepOnePlus system (Applied Biosystems, Foster City, CA, USA) with specific primers (forward, 5′-GCGTCAGTAACAGCCCAGAG-3′; and reverse, 5′-GCTTGTAGGCGGTTCGTC-3′) for *G. vaginalis*. The PCR conditions were as follows: 95 °C for 1 min, followed by 40 cycles of denaturation at 95 °C for 15 s, annealing at 55 °C for 15 s, and extension and fluorescent data collection at 72 °C for 45 s. For melting curve analysis, the temperature was decreased from 95 °C to 65 °C at a rate of 0.1 °C/s, with the continuous acquisition of the fluorescence signal intensity. The data were analyzed using the Applied Biosystems software. The percentage inhibition rate in the co-culture was calculated using the following formula: % inhibition rate = (1 − 1/2^ΔΔCt^) × 100. The experiments were performed twice in triplicate.

### 2.6. Evaluation of Lactobacillus sp. for Antagonism of G. vaginalis Adhesion

The antagonistic effects of the probiotics on *G. vaginalis* adherence to HeLa cells were assayed according to a method described by Pessoa et al. [25], with slight modifications. HeLa cells (1 × 10^8^ cells/mL in 1 mL of DMEM) were incubated in 12-well culture plates for 24 h, followed by incubation with *Lactobacillus* sp. (at 10^5^, 10^7^, 10^9^ CFU/mL suspended in DMEM) or vehicle at 37 °C in a cell incubator for 3 h. The initial concentration of *G. vaginalis* was 10^4^ CFU/mL in the co-culture assay because *G. vaginalis* will grow in BHIs broth after a 24-h co-culture. The concentration of *G. vaginalis* used in the adhesion test was 10^7^ CFU/mL, owing to the fact that the adhesion test only lasted 3 h. The growth of the *G. vaginalis* can be ignored. The plates were then washed twice with PBS, and *G. vaginalis* (1 × 10^7^ CFU/mL suspended in DMEM) was added and incubated at 37 °C for another 3 h. The plates were then washed three times with PBS, and the inhibition rate of *G. vaginalis* attachment to the cells was assayed using the qPCR method described in [29].

### 2.7. Effects of LAB CFS on G. vaginalis Biofilm Formation and Preformation

The effects of *Lactobacillus* sp. CFS on biofilm formation was evaluated according to the methods of [30], with slight modifications. The CFS was prepared using the same procedure used for SCFA analysis. A 100-µl aliquot of the CFS was added to 100 µL BHIs broth containing 10^5^ to 10^6^ CFU of *G. vaginalis*, with the control sample prepared by adding 100 µl MRS broth instead of CFS. A 200-µl volume of each sample was then transferred to a 96-well microtiter plate, following incubation at 37 °C for 48 h in an anaerobic chamber (Ruskinn Technology, Ltd.). To assess the extent of biofilm formation in each microplate, the culture medium was discarded, and the microplate was washed twice with 200 μL of PBS. The adherent biofilm cells were stained with 200 μL of 0.1% (*w/v*) Crystal Violet for 15 min and rinsed twice with PBS. After removing the bound dye from the stained cells with 200 μL of absolute ethyl alcohol, the amount of biofilm was quantified by measuring the OD_600_ of the solution with a spectrophotometer (BioTek, Winooski, VT, USA). There were five replicates prepared for each sample, and the experiment was performed in triplicate.

The assessment of the CFS effects on preformed *G. vaginalis* biofilm was performed according to the methods of [31], with small modifications. A *G. vaginalis* culture was performed in a 96-well microtiter plate with 200 μL BHIs broth prior to incubation at 37 °C for 48 h. The culture supernatant was removed, and the cells were washed twice with PBS. CFS (200 μL) was then added to each well prior to incubation at 37 °C for 24 h. Reductions in the biofilm formation were determined following the reference.

### 2.8. Impact of Lactobacillus sp. CFS on G. vaginalis Gene Expression

To investigate underlying anti-*G. vaginalis* mechanisms, we evaluated *G. vaginalis* genes in mRNA levels, which encode proteins related to virulence factors (*HMPREF0424_0103* (*vly*)*, HMPREF0424_1109* (*sld*)), epithelial adhesion (*HMPREF0424_0125* (*pat*)), biofilm formation (*HMPREF0424_0821* (*gtf*)), metabolism (*HMPREF0424_1297* (*stp*), *HMPREF0424_1253* (*atm*), and *HMPREF0424_1189* (*itm*)), and antimicrobial resistance (*HMPREF0424_0156* (*bcrA*) and *HMPREF0424_1122* (*mds*)), by reverse transcription PCR, according to the methods of [31]. *G. vaginalis* was grown at 37 °C for 48 h in BHI broth, after which 500 μL of *G. vaginalis* suspension and 500 μL of CFS were added to 4 mL of BHI broth and incubated anaerobically in an anaerobic system (Ruskinn Technology, Ltd.) at 37 °C for 48 h. In the control wells, the *Lactobacillus* sp. CFS were replaced with MRS broth. After incubation, the culture suspension was removed from the wells for RNA extraction from floating bacteria. Cells adhering to the plate wells were washed twice with sterile PBS, and the RNA was extracted from the biofilm. The total RNA was extracted using TRIzol (Beyotime, Beijing, China) according to manufacturer instructions, and the RNA concentration and purity were determined using a microplate reader (BioTek). The ReverTra Ace qPCR RT kit (Toyobo) was used to reverse transcribe 2 μg of total RNA into cDNA.

We used qPCR to examine the effect of *Lactobacillus* sp. on target gene expression in *G. vaginalis* according to the methods of Reham Wasf et al. [31]. The cDNA was amplified by real-time qPCR using the SYBR Green Real-Time PCR Master Mix on a StepOnePlus system, with the primers listed in Supplementary Table S1. We designed the primers according to the available literature and the complete genome of *G. vaginalis* 409-05. The PCR conditions were as follows: 95 °C for 1 min, followed by 40 cycles of denaturation at 95 °C for 15 s, annealing at 60 °C for 15 s, and extension and fluorescent data collection at 72 °C for 45 s. The data were analyzed using the Applied Biosystems software, and differences in the mRNA-expression levels were calculated after normalizing to the 16S rRNA level. The results are expressed as fold change compared to the control group based on the analysis of ΔΔCt values.

### 2.9. Anti-Inflammatory Effects of Probiotic Bacteria on HeLa Cells

The anti-inflammatory effects of probiotic bacteria on HeLa cells were evaluated according to the methods of [32,33]. *G. vaginalis* and *Lactobacillus* sp. (10^8^ CFU/mL of bacterial cells, final concentration) were resuspended in DMEM and incubated with HeLa cells for 3 h; with HeLa cells treated with DMEM for 3 h used as a control. The treated HeLa cells were washed twice with pre-warmed PBS (pH 7.0) and detached from the culture plates, after which TRIzol reagent (Beyotime) was added, and cells were stored at −80 °C until further use for RNA extraction.

The influence of probiotics on *G. vaginalis*-induced immunoreaction was evaluated according to the methods of [32], with minor changes. The HeLa cells with *G. vaginalis* (10^8^ CFU/mL) were incubated for 3 h in a 5% CO_2_ incubator, followed by the addition of probiotic cultures up to a final concentration of 10^8^ CFU/mL prior to incubation for another 3 h at 37 °C in a CO_2_ incubator. The cells were then treated with TRIzol reagent, as described, and untreated HeLa cells were chosen as a negative control.

Oxidative stress was induced in the HeLa cells by treatment with 0.5% H_2_O_2_ [32] prior to incubation in a 5% CO_2_ incubator for 3 h. Freshly grown probiotic bacteria (with a final concentration of 10^8^ CFU/mL) were resuspended in DMEM and added to each well and incubated for another 3 h. The HeLa cells treated with the probiotic bacteria were washed twice with pre-warmed PBS (pH 7.0) and detached from the culture plates, followed by collection, as previously described. RNA extraction and real-time PCR were performed as described in Section 2.8.

### 2.10. Statistical Analysis

The results were analyzed using the GraphPad Prism software (v.6.0; GraphPad Software, San Diego, CA, USA). All the results are expressed as the mean ± SD or the mean. A one-way analysis of variance was performed, and comparisons of the data for statistical significance were performed by either Dunnett’s multiple comparison test or Tukey’s multiple comparison test. Statistical significance was defined as *p* < 0.05 or fold change (≥ 2 or ≤0.5).

## 3. Results

### 3.1. Auto-Aggregation Ability, Hydrophobicity, Adhesion Ability to HeLa Cells and Survivability in 0.01% H_2_O_2_ of Three Candidate Strains

Firstly, we compared the probiotic properties of the candidate strains. The auto-aggregation ability and surface hydrophobicity of bacteria are two indirect methods for evaluating bacterial adhesion [34]. As shown in Table 1 and Supplementary Figure S1, *L. delbrueckii* DM8909 showed the strongest activity (51.5%), and the auto-aggregation rates of *Lpb. plantarum* ATCC14917 and *Lpb. plantarum* ZX27 were 11.65% and 8.10%, respectively. We observed moderate surface hydrophobicity in ATCC14917 and ZX27 (microbial adhesion to hydrocarbons (MATH) ∈ (33%−66%), whereas DM8909 exhibited low hydrophobicity (MATH < 33%) at 29.07%. HeLa cells were used to test the adhesion of *Lactobacillus* sp. to vaginal epithelial cells in vitro. DM8909 showed higher adhesion ability (35.96%) than *Lpb. plantarum* ATCC14917 (8.89%) and ZX27 (12.67%), with 593, 147, and 209 bacilli adhered per HeLa cell, respectively. Additionally, the survivability rates of DM8909, ATCC14917, and ZX27 in 0.01% hydrogen peroxide (H_2_O_2_) were 55.50%, 11.97%, and 91.12%, respectively. Overall, DM8909 exhibited superior performance of adherence and co-culture with *G. vaginalis*. However, *Lpb. plantarum* ZX27 showed superior adherence, co-culture, and oxidation resistance compared to *Lpb. plantarum* ATCC14917.

### 3.2. SCFAs and Lactic Acid Levels in CFS

SCFAs and lactic acid are important metabolites produced by *Lactobacillus* and *Bifidobacterium* and play an important role in maintaining intestinal health [35]. We analyzed the concentrations of six SCFAs and lactic acid in the CFS of cultured *Lactobacillus* sp. by gas chromatography to detect the differences in acid metabolites among candidate strains (Table 2). The other two *Lpb. plantarum* strains produced more lactic acid, propionic acid, butyric acid, isobutyric acid, and valeric acid than *Lactobacillus delbrueckii* DM8909. In addition, the concentrations of SCFAs and lactic acid in CFS of *Lpb. plantarum* ZX27 were found 0.003 mM (acetic acid), 0.106 mM (lactic acid), 0.032 mM (propionic acid), 0.060 mM (butyric acid), 0.014 mM (isobutyric acid), and 0.005 mM (valeric acid) higher than *Lpb. plantarum* ATCC14917. DM8909 produced slightly higher amounts of acetic acid and isovaleric acid than the other strains.

### 3.3. Interaction between Lactobacillus and G. vaginalis in Co-Aggregation, Co-Culture and Preventing Attachment

**Figure 1:**
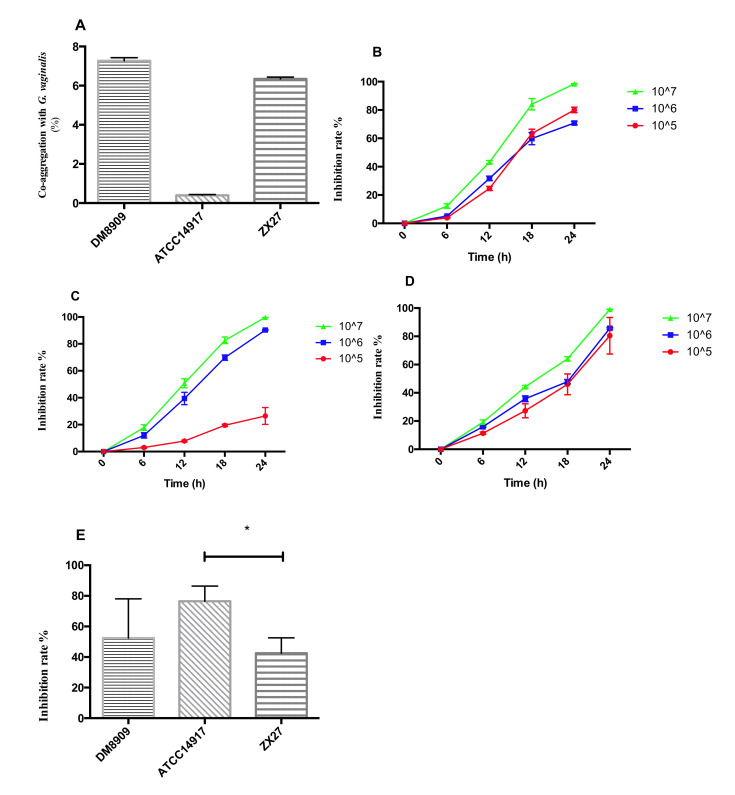
(**A**) Co-aggregation of three *Lactobacillus* sp. with *G. vaginalis.* Inhibition rates of *G. vaginalis* in a co-culture assay with (**B**) *L. delbrueckii* DM8909, (**C**) *Lpb. plantarum* ATCC14917, and (**D**) *Lpb. plantarum* ZX27. The initial concentration of *G. vaginalis* was 10^4^ CFU/mL. (**E**) Inhibition rate of *G. vaginalis* adhesion following pre-adhesion by *Lactobacillus* sp. (10^9^ CFU/mL). The initial concentration of *G. vaginalis* was 10^7^ CFU/mL. * *p* < 0.05. The data is represented as mean ± SD (*n* = 3).

Following 3 h of incubation with *G. vaginalis*, the co-aggregation value of the three lactobacilli ranged from 0.40% to 7.27%. Specifically, the co-aggregation ability of DM8909 (7.27%) and ZX27 (6.34%) was similar, whereas ATCC14917 showed poor co-aggregation (0.40%) (Figure 1A). The impact of the three lactobacilli on the growth of *G. vaginalis* was then evaluated in BHIs broth. A concentration series of 10^5^, 10^6^, and 10^7^ CFU/mL *Lactobacillus* sp. was used in the co-culture assays. As shown in Figure 1B–D, at a concentration of 10^5^ CFU/mL, the inhibition rates of DM8909, ATCC14917, and ZX27 were 80.06%, 26.44%, and 80.42%, respectively (24 h). Additionally, at 10^6^ CFU/mL, the inhibition rates of all three lactobacilli were 70.82%, 90.28%, and 85.72%, respectively. Furthermore, at 10^7^ CFU/mL, all three lactobacilli showed excellent anti-*G. vaginalis* abilities, with inhibition rates over 98%. Moreover, the suppressive effect of *Lactobacillus* sp. on *G. vaginalis* growth increased along with the increase in the number of *Lactobacillus* sp., with the biggest changes observed in ATCC14917 cells (ATCC14917 at 10^5^ CFU/mL showed an inhibition rate of 26.44%, as compared with 90.28% at 10^6^ CFU/mL) (Figure 1C). 

We then tested the protective effect of *Lactobacillus* sp. against *G. vaginalis* adhesion according to the competitive adhesion site of *Lactobacillus* sp. and *G. vaginalis* in HeLa cells. Preferential occupation of the adhesion site by *Lactobacillus* sp. would preclude the *G. vaginalis* attachment site, resulting in abortive binding to vaginal epithelial cells. The evaluation of the three lactobacilli concentrations (10^7^, 10^8^, and 10^9^ CFU/mL) revealed a protective effect only at the highest concentration (10^9^ CFU/mL, corresponding to 100-fold of the concentration of *G. vaginalis* used). The results show average inhibition rates for DM8909, ATCC14917, and ZX27 of 52.39%, 76.49%, and 42.57%, respectively (Figure 1E).

### 3.4. Impact of CFS on the Formation and Disruption of G. vaginalis Biofilms

**Figure 2:**
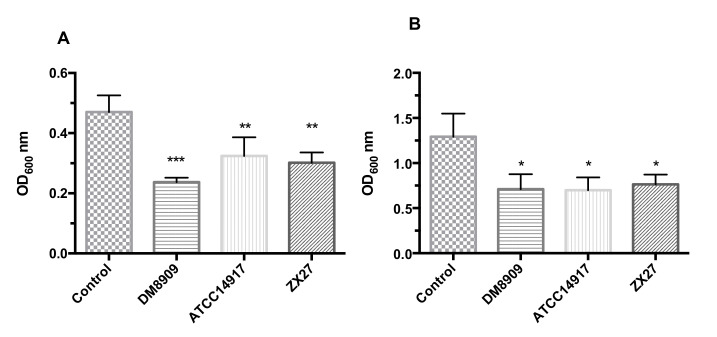
Impact of *Lactobacillus* sp. CFS on *G. vaginalis*. (**A**) Impact of CFS on *G. vaginalis* preformed biofilm. Optical density at 600 nm (OD_600_) of *G. vaginalis* biofilm in the presence of CFS. (**B**) Impact of CFS on *G. vaginalis* biofilm formation. Control: *G. vaginalis* growth in BHIs broth. Values are showed as mean ± SD (*n* = 5). *p < 0.05, **p < 0.01, ***p < 0.001 vs. control.

The impact of CFS produced by *Lactobacillus* sp. on *G. vaginalis* biofilm was also evaluated to further study the inhibition effect of *Lactobacillus* sp. The CFS of the three lactobacilli caused a significant reduction (*p* < 0.05) in *G. vaginalis* biofilm formation and preformed biofilm (DM8909: 49.55% and 44.91%; ATCC14917: 31.08% and 35.89%; and ZX27: 45.79% and 40.84%, respectively) (Figure 2A,B).

### 3.5. Impact of CFS in Gene Expression in Planktonic and Biofilm Cells of G. vaginalis

The CFS of the three LAB inhibit *G. vaginalis* growth and biofilm formation, according to the results of the present study and our previous work [24]. CFS contain *Lactobacillus* sp. metabolites, including SCFAs, H_2_O_2_, and bacteriocins [36]. In the present study, we used quantitative polymerase chain reaction (qPCR) to evaluate and compare the effect of LAB metabolites on *G. vaginalis* by exposing *G. vaginalis* to the CFS of the three *Lactobacillus* sp. (diluted 1:9 in BHIs) for 48 h. We compared the expression of nine genes from planktonic and biofilm-forming cells with that of untreated cells. The selected genes are involved in virulence systems (*vly* and *sld*), pili proteins (*pat*), biofilm formation (*gtf*), metabolism (*stp*, *atm,* and *itm*), and antimicrobial resistance (*bcrA* and *mds*).

Table 3 shows the fold changes in *G. vaginalis* at an mRNA level caused by the CFS of the three probiotic strains. DM8909 CFS upregulated three genes (*sld*, *gtf*, and *stp*) and did not impact the expression of the other six genes in planktonic cells. However, DM8909 CFS downregulated six genes (*vly*, *sld*, *gtf*, *stp*, *bcrA,* and *mds*), with no significant influence on the other three genes related to biofilm formation. ATCC14917 downregulated three genes (*gtf*, *bcrA*, and *mds*) in planktonic cells and seven genes (*vly*, *sld*, *pat*, *gtf*, *stp*, *bcrA*, and *mds*) in biofilm cells. Moreover, ZX27 significantly reduced the expression of all genes in both cell types, except for *bcrA* and *mds* in planktonic cells. These findings indicate that *Lpb. plantarum* ZX27 was the most effective for suppressing the expression of genes related to *G. vaginalis* pathogenicity.

### 3.6. Anti-Inflammatory Activities of Lactobacillus sp.

**Figure 3:**
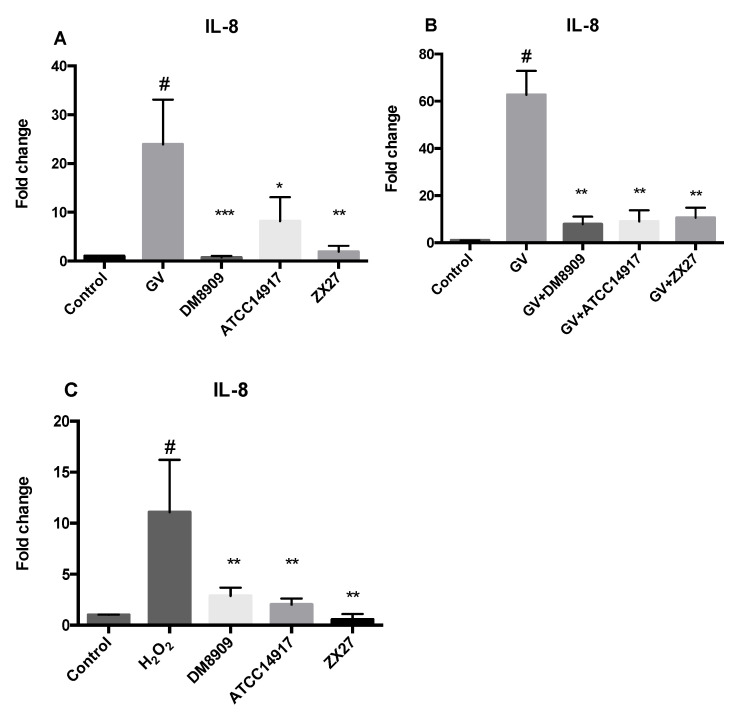
Regulation of pro-inflammatory IL-8 expression in HeLa cells by *Lactobacillus* sp. (**A**) IL-8 expression in HeLa cells simultaneously infected with *G. vaginalis* ATCC49145 (GV) and exposed to *Lactobacillus* sp. (**B**) IL-8 expression in HeLa cells infected with *G. vaginalis* ATCC49145 (GV), followed by *Lactobacillus* sp. treatment. (**C**) IL-8 expression under oxidative stress following treatment with *Lactobacillus* sp. Control: untreated HeLa cells. Data represent the mean ± SD (*n* = 3). ^#^*p* < 0.05 vs. Control; ^*^p < 0.05, ^**^p < 0.01, ^***^p < 0.001 vs. GV or H_2_O_2_ alone.

We then assessed the effects of different *Lactobacillus* sp. strains on interleukin (IL)-8 production in HeLa cells. We found that *G. vaginalis* could significantly induce IL-8 expression; nevertheless, *Lactobacillus* sp. strains induced IL-8 expression less than *G. vaginalis* (*p* < 0.05) (Figure 3A). Additionally, IL-8 mRNA levels in HeLa cells exposed to the three *Lactobacillus* sp. strains did not differ significantly from those in the negative control (*p* > 0.05).

Moreover, we observed that the upregulated IL-8 expression in HeLa cells induced by *G. vaginalis* was significantly downregulated following subsequent post-treatment with probiotic *Lactobacillus* sp. (*p* < 0.01) (Figure 3B).

To further evaluate the anti-inflammatory activity of *Lactobacillus* sp. under oxidative stress, HeLa cells were exposed to H_2_O_2_ [32], which upregulated IL-8 expression. However, the following subsequent post-treatment with the three lactobacilli significantly reduced IL-8 expression in HeLa cells, compared with the H_2_O_2_ group (*p < 0.05*) (Figure 3C).

## 4. Discussion

BV is among the most common vaginal conditions in women of child-bearing age worldwide. Optimal vaginal microbiota comprises a balanced mixture of more than 250 bacterial species [37]. Under certain conditions, changes in vaginal microbiota are always accompanied by a decrease in *Lactobacillus* sp. and an overgrowth of anaerobic bacteria. *G. vaginalis* is a major contributor to BV due to its virulence factors, including vaginolysin and sialidase [9,13]. *Lactobacillus* sp. have been proved to efficiently treat BV and reduce its recurrence by restoring vaginal microbiota [19,37,38,39,40].

*Lactobacillus* sp. exhibit antibacterial activity in different ways [25]. In the present study, we evaluated the probiotic ability and anti-inflammatory effects of three candidate lactobacilli and the pathways related to *G. vaginalis* adhesion, biofilm formation, and gene expression. *L. delbrueckii* DM8909 was a commercial strain isolated from Dingjunsheng (a live *Lactobacillus* capsule for vaginal use; Wanze Shuangqi). Our previous study confirmed *Lpb. plantarum* ZX27′s anti-*G. vaginalis* activity [24], and in this report, *Lpb. plantarum* ATCC14917 was used as a control for *Lpb. plantarum* ZX27.

We initially compared the basal properties of the three lactobacilli. The auto-aggregation capacity is a crucial factor that allows probiotic strains to form floccules under adverse conditions [41]. Two *Lpb. plantarum* strains isolated from cocoa fermentation showed moderate auto-aggregation values of 33.44% and 29.23% [25], and Yanfeng et al. [42] reported 15 *Lpb. plantarum* strains showing auto-aggregation values ranging from 24.83% to 33.57%. In the present study, *L. delbrueckii* DM8909 showed a notable auto-aggregation capacity of up to 51.5%, whereas *Lpb. plantarum* ATCC14917 and ZX27 showed lower capacities of 11.65% and 8.1%, respectively. It is likely that the auto-aggregation capacities of the two *Lpb. plantarum* strains in this study were inferior to those previously reported due to the different time periods evaluated. However, DM8909 had a lower MATH (<33%) according to hydrophobicity testing, which was consistent with previous findings [42]. Moreover, we assessed the adhesion abilities of the three strains to HeLa cells in saturation coverage (over 1000 strains on one HeLa cell, 10^9^ CFU *Lactobacillus* sp. strains covered 10^6^ HeLa cells). The number of *Lactobacillus* sp. to HeLa cells indicated the maximum number of *Lactobacillus* sp. on one cell (>100), suggesting that all three strains possessed notable adhesive properties. Furthermore, ZX27 showed better survivability under oxidative stress, and the results reveal significantly enhanced resistance to 0.01% H_2_O_2_ when compared with ATCC14917. Petrova et al. [27] tested three vaginal probiotic *Lactobacillus* sp. and found the survival rates of *Lactobacillus rhamnosus* GG, *L. rhamnosus* GR-1, and *L. rhamnosus* LC705 in 0.1% (*v/v*) H_2_O_2_ ranging from 1.8% to 8.7%. Additionally, we found that the probiotic properties were highly strain-specific, with ZX27 exhibiting moderate properties in hydrophobicity and adhesion. Bacterial colonization and adhesion are likely influenced by auto-aggregation [3]. Although ZX27 exhibited relatively poor auto-aggregation, it exhibited faster growth and a higher number of CFUs at the end of fermentation (12 h) (4 × 10^9^ CFU/mL) compared to DM8909 (5 × 10^8^ CFU/mL) (Supplementary Figure S2).

The evaluation of the SCFAs and lactic acid in the CFS of ZX27 indicated that it produced the highest content of lactic acid (8.8% higher than DM8909), propionic acid, butyric acid, isobutyric acid, and valeric acid. A low, acidic pH is generally considered to be a primary mechanism for maintaining the composition of healthy vaginal microflora [43]. Moreover, BV is characterized by a dramatic loss of lactic acid and greater concentrations of mixed SCFAs, including acetate, propionate, butyrate, and succinate [44]. In the present study, we found that ZX27 was a more appropriate candidate for BV therapy since this strain produced higher concentrations of propionic acid, butyric acid, isobutyric acid, valeric acid, and lactic acid than DM8909. 

In the co-aggregation test, DM8909 and ZX27 had a significantly higher co-aggregation ability with *G. vaginalis* than ATCC14917 (Figure 1A). Additionally, the observed co-aggregation with *G. vaginalis* in the present study (0.40–7.27%) was lower than previous reports (43.15–44.61%) [25]. The co-culture experiments showed that the original content of *Lactobacillus* sp. influenced the inhibition ability. With the content of 10^7^ CFU/mL, all three lactobacilli efficiently inhibited *G. vaginalis* growth (inhibition rate >98%) (Figure 1B–D). These results were consistent with those of other studies, including *Lpb. plantarum* Os13 and *Lpb. plantarum* Kor14 inhibition of *Listeria monocytogenes*, *E. coli*, and *Salmonella enterica* serovar Enteritidis growth at different times with an initial *Lactobacillus* sp. content of 10^7^ CFU/mL [45]. Additionally, *Lactobacillus johnsonii* LJ202 inhibited the growth of *S. enterica* serovar Enteritidis DMST7106 in a co-culture study [46], and another study reported that a 4-h co-culture with *Lactobacillus gasseri* eliminated *G. vaginalis* and *Prevotella bivia* [47]. Moreover, two commercially available probiotic strains (*L. rhamnosus* HN001 and *Lactobacillus acidophilus* GLA-14, alone or in combination (Respecta probiotic blend)) inhibit the growth of four different pathogens responsible for both BV (*G. vaginalis* and *Atopobium vaginae*) and aerobic vaginitis (*Staphylococcus aureus* and *E. coli*) in co-cultures [48]. Furthermore, *Lpb. plantarum* 86 and *L. fermentum* AI2 inhibit the growth of *E. coli* NG 502121 and *S. aureus* AY 507047 following co-culture [49]. We found that *Lactobacillus* sp. inhibited *G. vaginalis* adherence when HeLa cells were previously covered with *Lactobacillus* sp. (Figure 1E) and ATCC14917, showing the strongest inhibition to prevent *G. vaginalis* adherence. This result contrasts with reports indicating poor adherence of ATCC14917 to HeLa cells (Table 1). The stronger adhesive ability of ATCC14917 might be one of the possible explanations, and the poor co-aggregation ability of ATCC14917 with *G. vaginalis* may explain the higher adhesion inhibition rate of this *Lactobacillus* strain (Figure 1A,E). At a lower *Lactobacillus* sp. concentration of 10^5^ CFU/mL, *G. vaginalis* only showed good growth in a co-culture with ATCC14917. This might be related to the poor co-aggregation ability of ATCC14917 with *G. vaginalis* (Figure 1A,C). The poor co-aggregation ability of *Lactobacillus* resulted in a weak anti-pathogens ability with respect to a low probiotic number but provided stronger mucosal protection in the adhesion test. A previous study showed that *L. crispatus* significantly inhibits *G. vaginalis* adherence [21], and *Lactobacillus* sp. were capable of excluding and displacing urinary tract pathogens from SV-HUC-1 cells [50]. Additionally, Liu et al. [51] found that *Lpb. plantarum* CCFM 233 and *Lpb. plantarum* CCFM 231 strongly antagonized the adhesion to and invasion of HT-29 cell lines by enteroinvasive *E. coli*.

In addition to the antimicrobial activity of the three lactobacilli, we discovered that their respective CFS exhibited anti-biofilm activity against *G. vaginalis*. Mariya et al. [52] reported that the lectin-like protein 1 in *L. rhamnosus* GR-1 inhibits adhesion and biofilm formation in the key urogenital pathogen *E. coli* UTI89. In addition to its anti-biofilm formation activity, we found that the CFS destroyed *G. vaginalis* biofilm. This was supported by a previous study showing that *L. rhamnosus* GR-1 and *L. reuteri* RC-14 infiltrate *G. vaginalis* biofilms and cause bacterial cell death [53]. *G. vaginalis* biofilms tolerate 5- and 4-8-fold higher concentrations of H_2_O_2_ and lactic acid, respectively, than planktonic cultures [53]. In the present study, we demonstrated that *Lactobacillus* sp. were capable of eliminating biofilm and efficiently killing *G. vaginalis*.

To investigate the mechanism by which *Lactobacillus* sp. antagonizes *G. vaginalis*, we evaluated the expression of relevant genes encoding virulence factors and their association with adhesion, biofilm formation, metabolism, and antimicrobial resistance (Table 3). The *vly* and *sld* genes encode vaginolysin and sialidase, which are the main pathogenic factors of *G. vaginalis*. We found that *vly* and *sld* expression was downregulated in both planktonic and biofilm cells by the CFS of ZX27, indicating that *Lactobacillus* sp. CFS influences virulence factors produced by *G. vaginalis*. It is clear that *Lactobacillus* sp. metabolites affected the expression of pathogenic genes. A previous study demonstrated that *L. crispatus* directly downregulated the expression of *vly* in *G. vaginalis* UM241, a BV-positive strain [9]. Furthermore, exposure to CFS decreased the expression of *pat*, which encodes the fibril-associated protein (flp) pilus-assembly TadE/G-like family protein and might play an important role in its adhesion to vaginal epithelial cells [54]. These findings suggest that *Lactobacillus* sp. CFS might decrease the package of pilus in *G. vaginalis* to inhibit adhesion. Deconvolution microscopy showed changes in the structure and viability of biofilms in the presence of *L. reuteri* RC-14, accompanied by the loss of dense *Gardnerella* biofilm pods [22]. The *gtf* gene encodes a glycosyltransferase, which is likely imperative for the biosynthesis of exopolysaccharide and critical for biofilm formation [54]. Except for DM8909, we found that CFS decreased the expression *gtf* in *G. vaginalis*. The *stp*, *atm*, and *itm* genes of *G. vaginalis* are involved in the metabolism of carbohydrates, amino acids, and ions, respectively (the function of these three genes are in Supplementary Table S1). In the present study, we found that ZX27 CFS decreased the expression of these genes, suggesting that *Lactobacillus* sp. CFS inhibited *G. vaginalis* metabolism. Furthermore, the expression of *bcrA* and *mds* involved in antimicrobial resistance was downregulated in biofilm cells exposed to the CFS from all three lactobacilli. Interestingly, only ATCC14917 CFS downregulated *bcrA* and *mds* expression in planktonic cells. In our previous study, *Lactobacillus* sp. directly inhibited *bcrA* and *mds* expression [24]. This phenomenon might explain the better potency of probiotics combined with antibiotics for BV therapy compared to antibiotics alone [5]. Castro et al. [54] used RNA-seq technology to reveal upregulated expression of *pat*, *gtf*, *bcrA,* and *mds* in biofilm cells as compared with planktonic cells. In the present study, we found that *Lactobacillus* sp. decreased the expression of biofilm-related genes in *G. vaginalis* and inhibited biofilm formation, agreeing with a previous study showing that lactobacilli infiltrate BV biofilms and result in bacterial death [53]. Notably, the CFS from DM8909 and ATCC14917 upregulated *sld* expression by 10-fold in planktonic cells, and DM8909 also upregulated *gtf* and *stp* in suspended *G. vaginalis* cells. By contrast, these three genes were downregulated by DM8909 CFS in biofilm cells, with this downregulation also observed in *sld* and *pat* by ATCC14917 CFS. A possible explanation is that *G. vaginalis* responds differently to *Lactobacillus* sp. CFS in suspended and biofilm states. The CFS of *L. delbrueckii* DM8909 upregulated *sld*, *gtf,* and *stp* genes of *G. vaginalis* in planktonic form. One possible explanation is that free pathogenic bacteria prefer to make pathogenicity, while bacteria in the biofilm are more likely to survive in the presence of CFS from *L. delbrueckii* DM8909. This represents the first study reporting an altered expression of *G. vaginalis* genes associated with adhesion (*pat*), biofilm formation (*gtf*), metabolism (*stp*, *atm*, and *itm*), and antimicrobial-resistance (*bcrA* and *mds*) by *Lactobacillus* sp.

We also evaluated the effect of *Lactobacillus* sp. on IL-8 production. Previous studies reported that *E. coli*-induced production of IL-8, a pro-inflammatory cytokine, is inhibited by *Lactobacillus* sp. in HT-29 cells [33], and *Lpb. plantarum* ATG-K2, ATG-K6, and ATG-K8 lysates decrease lipopolysaccharide-induced production of IL-6 and tumor necrosis factor-α in murine macrophages [55]. In the present study, *Lactobacillus* sp. inhibited IL-8 production induced by *G. vaginalis* and H_2_O_2_. These results agree with a previous study showing that *Lactobacillus* sp. strains or *Lpb. plantarum* 59 and *L. fermentum* 137 CFS decreased IL-8 secretion in HeLa cells induced by *G. vaginalis* infection [23]. Additionally, Yang et al. [56] reported that *Lpb. plantarum* 200655 isolated from kimchi showed radical scavenging activity and lipid peroxidation-inhibition activity. In the present study, three lactobacilli displayed modulatory effects on IL-8 levels under oxidative stress conditions in HeLa cells. Further research on the anti-inflammatory function of *Lactobacillus* sp. in the vaginal environment is needed.

There are still some limitations in our studies. The adhesion and anti-inflammatory assays were performed in HeLa cells, which are a type of cancer cell; therefore, an assessment of these findings using normal vaginal epithelial cells may be more credible. Although experimental studies in mice have confirmed that *Lactobacillus* sp. can treat BV caused by *G. vaginalis* [29,57], additional studies in other animal models and humans are needed to fully explore the mechanisms and safety of vaginal-use probiotics.

In conclusion, we demonstrated that *Lactobacillus* sp. displayed four attributes related to anti-*G. vaginalis* activity: (1) growth inhibition, (2) adherence reduction via barrier function, (3) inhibition of anti-biofilm formation and attenuation of virulence factor expression, and (4) anti-inflammatory properties. Specifically, we showed that *Lactobacillus* sp. metabolites affected the expression of key genes, which is related to virulence factors, adhesion, biofilm formation, metabolism, and antimicrobial resistance in *G. vaginalis*. Multiple strains of *Lactobacillus* sp. may be used together for establishing vaginal eubiosis due to the specificity of probiotic strains. Our results identified *Lpb. plantarum* ZX27 as the possible candidate for further studies due to its satisfactory anti-*G. vaginalis* activity.

## Figures and Tables

**Table 1:** Auto-aggregation ability, hydrophobicity, and adhesion ability to HeLa cells, and survivability in 0.01% H_2_O_2_ of three candidate strains. (mean ± SD, *n* = 3).

	Auto-Aggregation Ability (%)	Hydrophobicity (%)	
Strain			
*L. delbrueckii* DM8909	51.50 ± 4.10	29.07 ± 0.45	
*Lpb. plantarum* ATCC14917	11.65 ± 1.49	50.97 ± 0.64	
*Lpb. plantarum* ZX27	8.10 ± 0.28	44.07 ± 0.15	
	**Adhesion to HeLa cells**		**Survivability in 0.01% H_2_O_2_ (%)**
**Strain**	Adhesion rate (%)	Amount of lactobacilli in each HeLa cell	
DM8909	35.96 ± 6.81	593 ± 112	55.5 ± 2.55
ATCC14917	8.89 ± 1.03	147 ± 17	11.97 ± 9.53
ZX27	12.67 ± 0.78	209 ± 13	91.12 ± 6.06

**Table 2:** Concentrations of short-chain fatty acids (SCFAs) and lactic acid in the cell-free supernatants (CFS) of *Lactobacillus* sp. cultured under anaerobic conditions. (mean ± SD, *n* = 3).

Strain	SCFAs and Lactic Acid (mM)						
	Acetic Acid	Lactic Acid	Propionic Acid	Butyric Acid	Isobutyric Acid	Valeric Acid	Isovaleric Acid
DM8909	0.012 ± 0.001	6.513 ± 0.034	0.951 ± 0.005	0.977 ± 0.003	0.611 ± 0.002	0.431 ± 0.002	0.285 ± 0.011
ATCC14917	0.008 ± 0.001	6.954 ± 0.031	1.021 ± 0.003	1.06 ± 0.004	0.654 ± 0.005	0.464 ± 0.003	0.283 ± 0.008
ZX27	0.011 ± 0.001	7.06 ± 0.064	1.053 ± 0.003	1.12 ± 0.021	0.668 ± 0.008	0.469 ± 0.001	0.282 ± 0.007

**Table 3:** Fold changes in the gene expression of *G. vaginalis* following exposure to *Lactobacillus* CFS. (Mean, *n* = 3).

Category	Target Gene (Symbol)	Fold change in Suspended Bacteria^a^			Fold Change in Biofilm^b^		
		DM8909	ATCC14917	ZX27	DM8909	ATCC14917	ZX27
Pathogenic factor	*HMPREF0424_0103* (*vly*)	1.41	1.21	0.15*	0.17*	0.16*	0.14*
	*HMPREF0424_1109*(*sld*)	45.33*	10.33*	0.05*	0.25*	0.25*	0.01*
Epithelial adhesion	*HMPREF0424_0125* (*pat*)	1.20	2.65*	0.02*	0.57	0.36*	0.0001*
Biofilm formation	*HMPREF0424_0821* (*gtf*)	2.08*	0.29*	0.07*	0.27*	0.25*	0.17*
Metabolism	*HMPREF0424_1297* (*stp*)	2.21*	1.57	0.17*	0.38*	0.11*	0.04*
	*HMPREF0424_1253* (*atm*)	1.51	0.87	0.19*	2.08*	0.89	0.17*
	*HMPREF0424_1189* (*itm*)	1.87	0.97	0.08*	0.72	0.72	0.07*
Antimicrobial resistance	*HMPREF0424_0156* (*bcrA*)	0.77	0.47*	1.64	0.10*	0.10*	0.30*
	*HMPREF0424_1122* (*mds*)	0.93	0.45*	1.83	0.17*	0.17*	0.39*

^a^ Control: *G. vaginalis* supernatant cultured in BHIs for 48 h; ^b^ Control: *G. vaginalis* cultured in BHIs for 48 h and adhered to a cell culture dish; * Denotes a statistically significant fold change (≥ 2 or ≤0.5).

## Data Availability

All datasets generated for this study are included in the manuscript and/or the Supplementary Files.

## Supplementary Materials

The following are available online at https://www.mdpi.com/article/10.3390/microorganisms9040728/s1, Supplementary Table S1: Primer sequences used for qPCR assay, Supplementary Figure S1: Time-dependent auto-aggregation rate of the three tested *Lactobacillus* sp., Supplementary Figure S2: The growth curve of the three tested *Lactobacillus* sp.
