# Supplementary material for: Probiotic Lactobacillus sp. Strains Inhibit Growth, Adhesion, Biofilm Formation, and Gene Expression of Bacterial Vaginosis-Inducing Gardnerella vaginalis
Source: Microorganisms. 2021 Mar 31;9(4):728. doi: 10.3390/microorganisms9040728 (PMC8065998; doi:10.3390/microorganisms9040728)
Supplement: Supplementary file 1 [file microorganisms-09-00728-s001.pdf]

Probiotic *Lactobacillus* sp. strains inhibit growth, adhesion, biofilm formation and gene expression of bacterial vaginosis-inducing *Gardnerella vaginalis*

Zhixiang Qian[1,2,3], Hui Zhu[1], Dan Zhao[1], Ping Yang[1], Fei Gao[1], Chunyi Lu[1], Yu Yin[1], Shidong Kan[1], and Daijie Chen[1,2*]

Supplementary Table 1 Primer sequences used for RT-qPCR assay.

[a]*vly*, HMPREF0424_0103, encoding athiol-activated cholesterol-dependent cytolysin, vaginolysin; *sld*, HMPREF0424_1190, encoding glycosyl hydrolase family 25, sialidase; *pat*, HMPREF0424_0125, encoding flp pilus-assembly TadE/G-like family protein; *gtf*, HMPREF0424_0821, encoding glycosyltransferase, group 2 family protein; *stp*, HMPREF0424_1297, encoding sugar ABC transporter permease; *atm*, HMPREF0424_1253, encoding ABC transporter permease; *itm*, HMPREF0424_1189, encoding glycosyltransferase, group 2 family protein; *bcrA*, HMPREF0424_0156, encoding bacitracin transport ATP-binding protein BcrA; *mds*, HMPREF0424_1122, encoding Multidrug resistance ABC transporter; 16s GV rRNA, 16s ribosomal RNA gene sequence.

[b]16s gene was used as an internal control in the Gardnerella vaginalis gene experiment.

[c]IL-8, interleukin-8

[d]GAPDH, glyceraldehyde-3-phosphate dehydrogenase, was used as the housekeeping gene

[e]Fw, Forward primer, Rv, Reverse primer

Target Gene[a]	Primer sequence (5' to 3')[e]	T $_{melting}$ (ºC)	Amplicon size (bp)
vly	Fw CTCGCATGCAGTACGATTCT<br>Rv TCTGGTGCATCAACGCTTAC	58<br>58	187
sld	Fw GGGTTTATGCACACGCTTTT<br>Rv GAAAATGCAGACAACGCAGA	56<br>58	131
pat	Fw GGTTCTGGCACTATGCTTGG<br>Rv ACACGCATTATCCTCCATCC	58.9<br>57.45	171
gtf	Fw CAACGAAGGCATAGGTTTCC<br>Rv GCGCTTGGAACTGCTTTAAC	59.57<br>60.02	156
stp	Fw TGGCTGTTATTGCTATCTACTTCA<br>Rv CTTCCAGAATACTTGCCACTTTGT	57.1<br>59.9	203
atm	Fw AGCAGCAATAACTGTAATGGTAATA<br>Rv CAGACTTTTCTTCTCTAGCAACTCC	56.1<br>58.5	155
itm	Fw AGTGCTGCTGCCGTGCTAT<br>Rv TCTGGAAGTGCCGCCTTAA	59.1<br>59.1	75
bcrA	Fw CCGACCGCATACCTATTTTG<br>Rv GCAAGACGGTCTCCAAACTC	60.34<br>59.85	178
mds	Fw CAGCACCTGTAGCTCCAACA<br>Rv TGGCTCAAGAGATTGTGTGC	60.05<br>59.99	195
16s GV rRNA[b]	Fw TGAGTAATGCGTGACCAACC<br>Rv AGCCTAGGTGGGCCATTACC	55.2<br>59.3	167
IL-8[c]	Fw TGGCAGCCTTCCTGATTTCT<br>Rv TTAGCACTCCTTGGCAAAACTG	59.3<br>59.38	61
GAPDH[d]	Fw AACGGATTTGGTCGTATTG<br>Rv GCTCCTGGAAGATGGTGAT	53.40<br>56.48	214

Supplementary Figure S1 Time-dependent auto-aggregation rate of the three tested *Lactobacillus* sp.

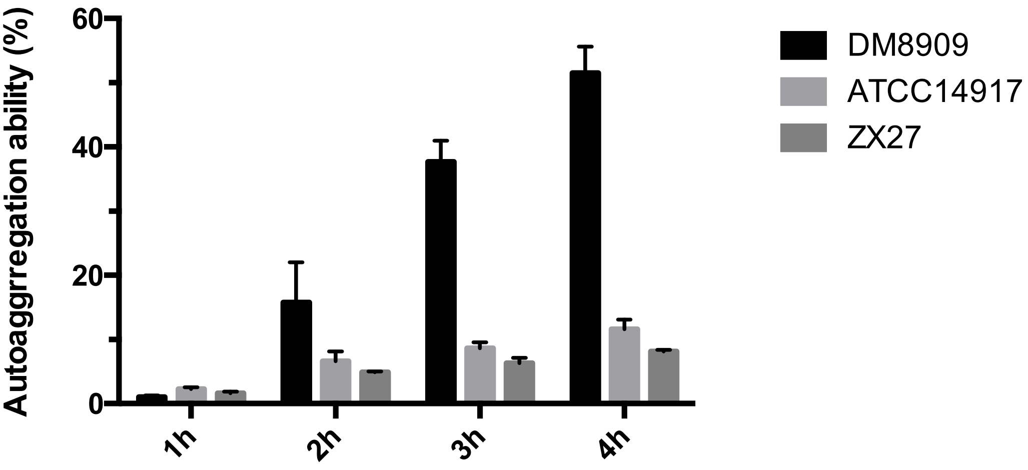

Data are expressed as the mean ± standard deviations (S.D) (n=3).

Supplementary Figure S2 The growth curve of the three tested *Lactobacillu* sp.

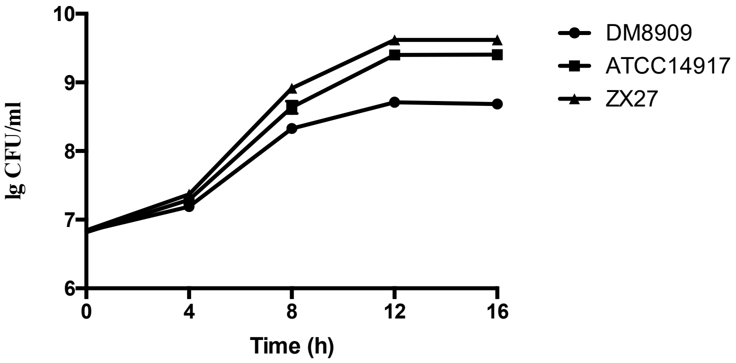

Data are expressed as the mean ± standard deviations (S.D) (n=3).
